# Cardiovascular Sciences Research Consortium Think Tank: Remote Monitoring for Cardiac Safety in Clinical Trials

**DOI:** 10.1016/j.jacadv.2025.102059

**Published:** 2025-08-08

**Authors:** Krishna Pundi, Sanjeev Bhavnani, Rosalyn Adigun, Jose Vicente, Rajesh Ghosh, David Albert, Waqaas Al-Siddiq, Charles Benson, Antoniu Fantana, Jennifer C. Goldsack, Salim F. Idriss, Gregory Marcus, Pamela Tenaerts, Mintu P. Turakhia, Jonathan Seltzer

**Affiliations:** a Veterans Affairs Palo Alto Health Care System, Palo Alto, California, USA; b Department of Medicine, Stanford University School of Medicine, Stanford, California, USA; c Department of Cardiology, Scripps Clinic, La Jolla, California, USA; d Department of Cardiovascular Medicine, Mayo Clinic, Rochester, Minnesota, USA; e Translational Cures, Inc, Washington, DC, USA; f Novartis, Cambridge, Massachusetts, USA; g AliveCor Inc., Mountain View, California, USA; h Biotricity, Inc, San Francisco, California, USA; i Eli Lilly, Indianapolis, Indiana, USA; j The Digital Medicine Society, Boston, Massachusetts, USA; k Division of Pediatric Electrophysiology, Duke University Medical Center, Durham, North Carolina, USA; l Division of Cardiology, University of California, San Francisco, San Francisco, California, USA; m Medable, Inc., Palo Alto, California, USA; n Cardiovascular Safety Research Consortium, Durham, North Carolina, USA

**Keywords:** biomarker, clinical trial, digital health, regulatory science, remote monitoring, safety

## Abstract

Recent advances in remote monitoring technologies have empowered continuous tracking of physiologic parameters with high accuracy and granularity. However, there are important differences in the hardware, data processing, and feedback streams of individual devices which can impact the use of digital health technologies when evaluating safety in studies. This expert panel is the result of a think tank through a public–private partnership of the Cardiovascular Sciences Research Consortium and the U.S. Food and Drug Administration. The white paper discusses regulatory considerations for remote monitoring through digital health technologies to provide a framework for their use when evaluating safety or adverse events. It also provides practical recommendations and best practices on the implementation of these technologies into clinical trials through a scientific, technical, and operational lens. This manuscript does not constitute regulatory guidance.

## Highlights

• Remote monitoring is becoming key to clinical trial design with the potential to reduce study overhead, improve access to care, and uncover novel data signals.
• Individual digital health technologies can vary, and are most effectively employed by anchoring to known endpoints, defining usability principles, and setting clinical guardrails.
• Large-scale private-public partnerships to redefine a “remote” physiologic baseline and cardiac events may uncover early safety signals and improve patient outcomes.

Safety monitoring is a critical component of clinical trials to keep volunteer trial participants safe and to understand safety signals that arise from the administration of the investigational medical product. Each stakeholder in the study must collaborate to implement a remote safety monitoring system that can responsibly and thoughtful identify, document, and address adverse events. Real-time data monitoring may empower an important step forward, as it may capture emerging safety signals sooner than evaluation at predetermined intervals. In addition, remote data captures allow more flexibility in trial design and may allow for a broader clinical trial population.^1^

There has been substantial recent innovation in remote monitoring technology, facilitating longer and more accurate cardiac monitoring. In addition to lightweight technology that can perform continuous electrocardiogram (ECG) monitoring as long as 30 days,^2^ machine learning algorithms have facilitated automated interpretations that rival those of trained cardiologists.^3^ Consumer devices can now monitor total atrial fibrillation (AF) burden, which can play an important role in clinical decision-making surrounding AF.^4^ Wearable technologies have demonstrated dramatic growth in the ability to measure or infer physiologic variables such as blood pressure, sleep quality, or exercise capacity, and there has been increasing overlap in the measurement capabilities of consumer and prescription cardiac monitoring technologies.^5^

However, effective use and implementation of these technologies can be challenging. Photoplethysmography and wearable-based ECG measurements have shown powerful potential for the detection of AF.^6^, ^7^, ^8^ But when applied to large populations with varying skin tones, technology use patterns, disease prevalence, and data signal quality, false positive rates can rise^9^ with results ranging from emotional distress to unnecessary clinical interventions. Algorithms have been developed to automate measurement of the QT interval from remote monitoring devices,^10^ but they still require clinician overread due to real-world variability in ECG quality and the potentially deadly effects of even small variations in measurement.^11^

An organizing guideline is needed to identify specific monitoring capabilities, validate them, and then enact them into real-time monitoring for drug and device safety studies. To address this need, the Cardiovascular Sciences Research Consortium hosted a think tank on March 1, 2024, inviting perspectives from scientists, clinicians, regulators, and industry representatives from pharmaceutical and medical technology companies. The overarching goal of this expert panel is to provide 1) a digestible summary of pertinent regulatory guidance on the use of digital health technologies (DHTs) in clinical trials and 2) a series of best practices and initiatives on how remote monitoring can be appropriately validated and used to monitor cardiac safety in clinical trials.

## Current guidance and mandates for use of digital health technologies in clinical trials

This section will discuss how safety is assessed when using DHTs to monitor study participants during clinical trials, the data reporting requirements when using DHTs to adjudicate clinical outcomes and/or adverse events, and to outline key requirements for using DHTs in device and drug development trials. It is not the objective of this section to recommend any specific trial methodologies or specific DHTs that can be incorporated within the design of clinical trials. Herein, the information provided will apply to DHTs that meet the definition of a medical device (Federal Food Drug & Cosmetic Act 201[h]); however, the information can also apply to DHTs that are not devices when used in clinical investigations.

### Scope of use of DHTs in clinical trials

The use of DHTs within clinical trials encompasses a regulatory framework from planning and selecting DHT technologies, managing and analyzing data acquired from DHTs, and for identifying DHT-derived endpoints that are consistent with the study outcomes of interest. In December 2023, the U.S. Food and Drug Administration (FDA) issued a final guidance on “Digital Health Technologies for Remote Data Acquisition in Clinical Investigations” that focuses on the use of DHTs in remote data acquisition from clinical trial participants in nonclinical settings (ie, in home-based environments).^12^ This guidance outlines that when compared to intermittent trial visitations that are usually conducted in clinical settings, the use of DHTs may allow for more frequent or continuous data collection with data captured remotely from trial participants in their usual environments. In the context of cardiac monitoring, data from passive (eg, wearable or patch-based ECG monitoring device) or active participant interaction with the DHT (smartwatch or smartphone-based ECG device) can inform what physiologic parameters and changes may meet an endpoint of interest (eg, onset and/or burden of AF). Such data collected from participants in their real-world settings can advance an understanding of the effectiveness, safety, and risk of an intervention in question, and how disease states change and are quantified through remote monitoring during the trial follow-up.

### Selecting appropriate DHTs for clinical trial event monitoring

Although this framework serves as a foundation for the design and execution of clinical trials, when considering the use of DHTs, it is important to understand that the selection of a DHT must be suitable to address a clinical outcome, and that the DHT is “fit-for-purpose”.^12^ Fit-for-purpose means that the DHT has a sufficient level of validation to support its use and interpretability in the clinical investigation and involves considerations of both the DHT’s form (ie, the design) and function(s) (that is, distinct purpose(s) within an investigation). In general, the intended use of a DHT in a clinical study may determine if the DHT is subject to investigational device exemptions regulations.^13^^,^^14^ In many cases, a DHT could be considered a nonsignificant risk or exempt and therefore would not require an investigational device exemptions application for use in a trial as a data collection medical device.

It is important to note that DHTs including software applications that qualify as a medical device are still subject to design control requirements which are basic controls to ensure that the devices will perform as intended. This applies to whether the DHT is used for studies whose primary purpose is to evaluate DHT devices and those that intend to use DHTs for data acquisition in clinical trials for other medical products.^14^ The FDA requires evidence of sufficient verification and validation for a DHT to be used for remote monitoring in a clinical investigation.^12^ This evidence is necessary to confirm the DHT is suitable for its intended data collection function (Table 1). Although a comprehensive discussion of the various specifications is beyond the scope of this section, provided in Table 1 is a summary of the key regulatory requirements for the use of DHTs for remote monitoring in clinical investigations. These recommendations also center on the need to improve representativeness of clinical trial populations and improve participation. In the aggregate, clearly describing the context of use, a clear rationale, and sufficient evidence to justify the use of DHTs ensures that regulatory considerations for the use of DHTs for remote monitoring in clinical trials of new products are broadly met. For study sponsors, the FDA encourages early engagement with the appropriate Center for feedback on whether a DHT poses significant risk, has sufficient validation for use, and is appropriate to a clinical investigation.

**Table 1:** General Summary of the Regulatory Considerations in the Digital Health Technologies for Remote Data Acquisition in Clinical Investigations Final Guidance

Regulatory Considerations	General Specifications for the Selection and Use of DHTs in Clinical Investigations
Selection of a DHT	The DHT used in a clinical investigation must meet acceptable feasibility and performance characteristics such as accuracy, precision, and consistency of measurements over time, and the uniformity of measurements across similar DHTs (eg, different brands of blood pressure devices used in different study subjects enrolled in the same clinical trial).
Study Participants	Identify the clinical benefits and risks including the context of use in a clinical trial (ie, how a study subject interacts with a DHT and how a study subject feels using a DHT).
Bring Your Own Device	Allowing trial participants to use their own DHTs may reduce the burden of using additional DHTs or other technologies used in a trial so long as the use of such device is fit-for-purpose.
Technical and Performance Specifications	Identify the minimum technical and performance specifications of the DHT (eg, model and/or version) for a DHT to remain fit-for-purpose. Ensure how DHTs capture the measurement(s) important to the disease or condition in question (eg, blood pressure, glucose, and ECG).
Verification	Verification is confirmation by examination and provision of objective evidence that the parameter that the DHT measures (eg, acceleration, ECG, pressure) is measured accurately and precisely.
Validation	Validation is the confirmation by examination and provision of objective evidence that the selected DHT appropriately assesses the clinical event or characteristic in the proposed participant population (eg, step count, heart rate and rhythm, and change in blood pressure).
Usability Evaluations of DHTs	Usability evaluations should assess the ability for trial participants to use the DHT in a remote setting without unacceptable errors so that measurements obtained from DHTs are accurate and precise.
Established Endpoints	Selecting or developing the clinical outcome assessment (eg, patient reported outcome, observer-reported outcome, clinical reported outcome, or performance outcome) that can be adequately measured and captured using DHTs (eg, blood pressure, weight, or ECG at home vs during an in clinical trial visitation).
Novel Endpoints	Justify whether a novel biomarker serving as a surrogate endpoint will reasonably predict a trial endpoint/outcome, and whether the effect of a trial intervention as captured by the novel endpoint is meaningful to the patient population for the medical product being evaluated.

DHT = digital health technologies.

#### Selection of DHTs appropriate to a target population

Consumer data have repeatedly shown that wearable technologies are disproportionately used by younger, higher-income individuals, with low incorporation among older and low-income groups.^15^ Separately, skin tone appears to reduce the accuracy of detection of heart rate and rhythm in some wearable devices,^16^ which warrants consideration when choosing a device for remote monitoring. In addition, even large pragmatic clinical trials have demonstrated enrollment of Black and Hispanic patients below their population representation, with disproportionate attrition of these individuals at each step of evaluation or follow-up.^8^^,^^17^ Children were generally excluded from the validation studies for most wearable monitors, and many of the variables measured by wearable monitors are either not FDA cleared for use in individuals ≤18 years of age or do not have published pediatric data.

#### Technical factors in the selection of DHTs

There are important differences in the hardware, processing algorithms, data storage, interpretation, and feedback streams for each of these monitors which can be clinically impactful when used to evaluate safety in studies.^2^ Acknowledging these limitations of remote monitoring technologies, to effectively incorporate them in clinical trials, they need standardized data streams, technological validation across populations, validation of use in the appropriate use cases, data quality checks, systems to capture adverse events, and systems to evaluate for falsified or fabricated data.

#### Privacy-related risks

In addition to existing requirements for patient data privacy, investigators and sponsors must consider unique privacy risks from using DHTs in clinical studies.^12^ Since there are additional risks of disclosure of identifiable participant information, measures such as modifying end-user licensing agreements or terms of services with data and computing platforms may be necessary on behalf of the DHT vendor. Additional attention should be paid to the security of data in transit between vendors and clinicians or investigators, and informed consent should discuss the added potential risks to patient security and how these risks are defrayed.

### Distinguishing digital endpoints from traditional clinical endpoints

Clinical safety is defined here as an untoward medical occurrence in a patient or clinical trial participant administered a product and which does not necessarily have to have a causal relationship with the treatment.^18^ Remote monitoring with DHTs can differ (based on use cases) from conventional safety monitoring processes due to the various participant and device factors listed above and in Table 1. In terms of data collection and data analysis,^19^ how digitally derived measurements identify and/or adjudicate an adverse event will depend on multiple additional analytical and clinical validation factors that include, but not limited to.

• Predefined data analysis methodologies (ie, the type of statistical methodologies to analyze frequent measurements over the duration of data collection and those that would account for day–night patterns, resting vs active, timing of measurements before and after a study intervention).
• Study participant compliance with remote monitoring schedules (ie, structured monitoring [eg, daily, weekly]) or capture of a monitoring parameter in a patient-reported fashion whenever needed, such as when symptomatic.
• Missing data resulting from data loss, study participant noncompliance, device connectivity, or filtering of a DHT monitoring parameter (eg, smart device captured AF) determined to be noise due to normal environmental conditions.
• Data interpretation and the reference ranges that accurately represent the clinical condition and adverse event within the population of interest.
• In scenarios where different brands of DHTs are used such as Bring Your Own Device clinical trials, standardized reference ranges and intervals of measurements may be required.

For example, frequent and serial blood pressure or patch-based ECG measurements in study participants may capture changes (ie, hypotension, cardiac arrhythmias) that represent the early identification of a safety signal or adverse event. However, such changes may be transient and quickly return to normal, especially in an asymptomatic participant. In such situations, it is unclear if the monitored parameter is a true or false identification of a predefined adverse event. In addition, study participants with a specific condition (eg, heart failure) may have a wide spectrum of disease severity (mild, moderate, and severe) and changes in remote monitoring parameters may result from a trial intervention, adjustments in heart failure related therapies, or the combination of both. As such, predefined data analyses that are used to quantify changes in study populations may not accurately capture individual or group effects that adjudicate an adverse event.^20^ Toward better understanding when a safety issue or adverse event occurs, DHTs used for remote monitoring should establish accurate patient-level normative values and those that can be used to quantify a change in the state of the condition in question. In doing so, DHTs may better identify the timing of an adverse event, facilitate the detection of a divergence from the preintervention baseline, and to more accurately identify when a safety concern arises.

## Best practices for remote monitoring in clinical trials

DHTs can be deployed at various stages of clinical trials from recruitment to study conduct to poststudy simulations/predictive modeling (such as through digital twins or risk prediction tools) (Central Illustration). In this section, we discuss important hurdles in the incorporation of DHTs for clinical trial safety monitoring, and propose best practices to support investigators, safety monitors, and sponsors. Proper implementation of novel technologies should be evaluated through an approach that addresses: 1) scientific issues—does the technology provide significant information to support regulatory grade evidence generation for cardiovascular (CV) endpoints?; 2) technical issues—was the technology properly evaluated for its intended use, and 3) operational issues—does it make sense to use the technology for the intended population and trial design. Using this approach, there are some core practical solutions which the clinical trial community can immediately adopt in its effort to improve detection and evaluation of CV events (Table 2).

**Central Illustration:**
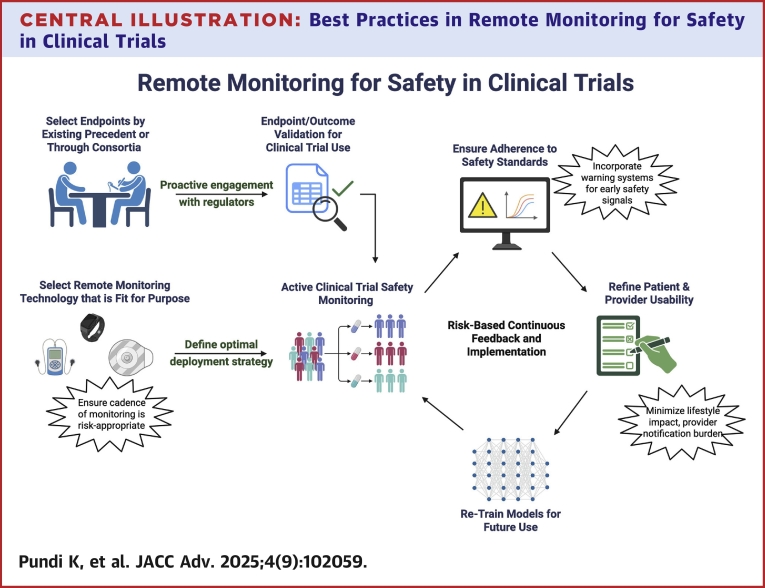
**Best Practices in Remote Monitoring for Safety in Clinical Trials** Remote monitoring and digital health technologies hold powerful potential for clinical trials and need to be deployed in a way that ensures patient safety while serving all stakeholders. Investigators and sponsors must identify technologies that are fit for purpose and determine whether they are appropriately verified or validated to the endpoints being measured. Technology that incorporates AI or prediction models must be trained appropriate to a target population, and the results of completed studies should be leveraged to retrain and improve models. During clinical trial deployment, investigators should build feedback loops for safety signals and ensure periodic evaluation of timeliness of responses. Finally, investigators must evaluate the impact of technologies on patients and clinicians both during and after the study to minimize lifestyle impact and notification burden. Created in BioRender. Pundi, K. (2025) https://BioRender.com/o962a0s. AI = artificial intelligence.

**Table 2:** Practical Use Cases for Remote Monitoring of Safety and Efficacy

Endpoint	Clinical Conditions	Necessary Test Characteristics	Role of Human in the Loop				Validation Requirement
			Screening	Efficacy	Safety	Post-Marketing	
Blood pressure	Hypertension Heart failure Chronic kidney disease Diabetes	1) Calibration to a reference (such as in-office machine) 2) Precision in measurement to detect changes	Minimal oversight	Feedback loop with near-term clinician involvement (24-48 hours)		AI-based systems with clinician available for review	Identify BP changes Determine association of BP trends to CV endpoints
Atrial fibrillation	Symptomatic atrial fibrillation Poststroke evaluation Postablation evaluation	High sensitivity through irregular rhythm/fast heart rate notifications for occurrence and evaluation of burden of AF	Determine if patient risk profile appropriate for study	Periodic evaluation (>72 hours) by clinicians	Feedback loop with near-term clinician response (24-48 hours)	AI-based systems and triage protocols with clinician available	Determine sensitivity & specificity with medical-grade devices, across populations Associate burden with CV endpoints
Corrected QT interval monitoring	Drug safety monitoring Arrhythmia management	1) High accuracy and precision in measurement 2) Calibration to reference	Determine if patient risk profile appropriate for study	Real-time monitoring with automated safety triggers to on-call clinician for rapid response (<8 hours)			Validation against large databases of 12-lead ECGs Association with CV endpoints

AF = atrial fibrillation; AI = artificial intelligence; BP = blood pressure; CV = cardiovascular; ECG = electrocardiogram.

### Digital CV clinical endpoint standards for detection of CV events

New technical approaches have the potential to generate data in less obtrusive ways. A core scientific question is whether the new technology establishes the presence or absence of a CV event to a suitable degree of regulatory certainty. Once agreed upon, these new approaches must be harmonized with extant endpoint definitions or used to create new endpoint definitions within the scientific community. Cardiovascular Sciences Research Consortium–like consortiums with academia, regulators, pharmaceutical companies, and technology providers have proved effective in this role^21^ and can play a key role in establishing standards which allow interchangeable use of different devices for the same purpose in different parts of the world, and seamlessly exchange data within the ecosystem. An approach could be to identify the measurement requirements for the endpoint of interest and define devices and technology factors that can collect the necessary measurements and appropriately characterize the data.

### Selecting and characterizing DHTs for use in clinical trials

As discussed above, a DHT must be “fit for purpose” to a clinical trial. To do this, investigators and sponsors should first define the objective and potential value of digital measurement or remote monitoring in a clinical trial. Depending on the protocol purpose, appropriate devices or DHTs may not have previously been validated in the specific context of the trials. However, the FDA does not require authorization from the Center for Devices and Radiological Health for a device to be used in clinical trials if it is safe for patient use (“nonsignificant risk”), because the FDA does not regulate the practice of medicine.

For some purposes, such as replacing a serum biomarker with a digital biomarker, the FDA requires traditional validation. In other situations, a DHT could be “characterized” through a structured pathway for the use in clinical trials. For instance, a DHT may be characterized as appropriate during clinical trial screening based on the data generated from its use in existing early-phase small clinical trials. A collaborative approach across sponsors and participating institutions with pooling of deidentified data could accelerate the DHT characterization and evidence generation. For investigators and sponsors, proactively collecting DHT data can facilitate characterization for the use in future trials.

### Cardiovascular endpoint validation for artificial intelligence, software as a medical device, and regulated medical devices

The FDA framework for the use of DHT in drug development^22^ provides guidance on validation and verification to ensure DHTs are accurate and reliable and meet intended users’ needs. Although verification is confirmation by examination and provision of objective evidence that the physical parameter that the DHT measures (eg, acceleration, temperature, and pressure) is measured accurately and precisely, validation is confirmation by examination and provision of objective evidence that the selected DHT appropriately assesses the clinical event or characteristic in the proposed participant population. As an example, a DHT that can monitor the heart rate in healthy individuals may not be applicable for users with heart failure or cardiac arrhythmias unless appropriately studied. Use cases for validation should be defined (ie, digital biomarker to support a cardiovascular efficacy primary endpoint) as well as suggesting acceptable approaches for validation (ie, the V3+ framework) for use of digital measures in a clinical trial. Utilizing and extending technical specifications to develop exploratory cardiac endpoints with prespecified analysis whenever possible will maximize mutual benefit and further refinement of such approaches.

### Processes for risk-based continuous implementation

Digital devices with embedded artificial intelligence (AI) may be trained and deployed where possible in settings with acceptable accuracy based on criticality of the biomarker/vitals. For instance, a device with higher sensitivity but lower specificity could be used to screen high-risk patients for longer hospitalization. On the other hand, if a device output is used for treatment decisions, it should be highly accurate. Deploying a device in a low-risk setting to “learn” using machine learning and human feedback approaches may be appropriate to enhance an AI algorithm in the absence of a large and clinically enriched training data set. Once the training is acceptable, the model needs to be frozen until the next planned iteration. Such an approach would allow the improvement of the device algorithm in parallel to creation of a large data set (during usage) for future training or prediction modeling. The FDA has developed the predetermined change control plan process to enable the continuous learning benefits of machine learning for medical devices. Overall principles are to ensure patient safety and enhance their outcome while encouraging innovation and flexibility.

### Usability principles which incorporate patient and health care provider perspectives

Although the development of novel devices should remain a priority, it is important to consider patient and health care provider burden resulting from inclusion in trials. For instance, multiple devices could be used to measure vitals and actigraphy as well as patient-reported outcomes. However, the burden of using multiple devices for an extended period could both exclude certain patients as well as impact their quality of life negatively. The burden of acting on multiple device alerts could also create an information overload for providers and negatively impact any decision-making processes. Careful consideration should be given to incorporate such perspectives to balance motivation, value, and burden whenever possible. A centralized repository for clinical outcomes and patient data across studies or across different arms could enable collection of measures from multiple devices. Different devices (within comparable range) could be treated as different labs for global trials to balance sensitivity and variability. Any use of AI could be copiloted with a human in the loop to refine machine learning approaches and use human error as the benchmark rather than 100% accuracy. Designing fit for purpose data collection and standards independent of capable technology could not only optimize cost/benefit but also the importance of device accuracy based on criticality of biomarker/vitals.

### Defining the proper role of technology–investigator interaction (ie, human in the loop)

One of the biggest appeals of digital technologies is the ability to passively or actively collect patient vitals more frequently and often continuously. This ability has the potential to alert patients and health care providers of any emerging safety risks (especially cardiac) and prevent worsening through early detection, improving overall patient outcomes. To ensure the safety of trial participants, periodic evaluation over the course of the study should ensure timely communication of potential safety events consistent with the trial protocol and regulatory approval.

Although digital technologies have the potential to generate unique insights, they do collect vast amounts of data from patients, automatic determination by the device without human in the loop^12^ has the potential to create noise/false positives whereby actual safety signals could be masked and the analysis burden may be increased. There is a need to develop a process whereby the need for “human in the loop” can be rigorously and consistently applied for detection of cardiovascular events in clinical trials.

## Conclusions

Here we discuss the pertinent and currently available guidelines on the use of remote monitoring for cardiac safety in clinical trials and propose a series of practical recommendations and initiatives applicable to both device and drug safety studies. Future research should be directed to “restudy” diseases such as AF in the context of new technologies. There is a need to redefine a baseline and define cardiac (adverse) events based on how naturalistic monitoring of vitals (ie, blood pressure or heart rate) correspond to or predict hard clinical endpoints/outcomes (ie, stroke) while balancing the risk of labeling an adverse outcome based on only slight perturbations in physiology.

AF burden, for instance, is an increasingly measured and available marker of disease. However, there is no standardization on approach of measurement, knowledge on the importance of daily or periodic trends, or sufficient clinical data on burdens and thresholds that warrant clinical intervention. Consortium approaches would help address the need for a large volume of standardized training data to develop AI based algorithms, promote collaboration on prioritizing, progressing and “validating” digital or “dynamic” biomarkers, generate longitudinal reference data, create a meaningful approach to trial subpopulations, and balance innovation with in vitro diagnostics regulatory approach for patient protection.

## Funding support and author disclosures

This Think Tank was supported by the Cardiovascular Sciences Research Consortium, a non-profit public-private partnership focused on advancing cardiovascular regulatory science. Dr Pundi has received research grants from the 10.13039/100000968American Heart Association, 10.13039/100005485American College of Cardiology, and the 10.13039/100000038Food and Drug Administration; consultant for iRhythm and Evidently. Dr Albert is an employee and founder of AliveCor, Inc. Dr Al-Siddi is an employee and founder of Biotricity, Inc. Dr Benson is an employee of Eli Lilly. Dr Fantana is an employee of Eli Lilly. Dr Goldsack is an employee of the Digital Medicine Society. Dr Marcus has received grant support from 10.13039/100000002NIH (10.13039/100000027NIAAA, 10.13039/100000050NHLBI, 10.13039/100000070NIBIB, 10.13039/100000026NIDA), the 10.13039/100000865Bill and Melinda Gates Foundation, the Patient Centered Outcomes Research Institute, the 10.13039/100005188Tobacco-Related Disease Research Program, and the California Department of Cannabis Control; consultant for InCarda; equity in InCarda. Dr Tenaerts is an employee and stockholder of Medable, Inc.; and has received honoraria for participation on data monitoring committee. Dr Turakhia has received research grants from 10.13039/100001009Bristol Myers Squibb, 10.13039/100000968American Heart Association, 10.13039/100004326Bayer, 10.13039/100005564Gilead Sciences, and the 10.13039/100000038Food and Drug Administration; consulting fees from 10.13039/100004374Medtronic, 10.13039/100004319Pfizer, 10.13039/100004331Johnson & Johnson; equity from iRhythm, Connect America, Forward, Evidently, PocketRN, AliveCor, Hippocratic.ai, Nuraxi Health; after the first think tank meeting, Dr Turakhia became an employee and corporate officer of iRhythm Technologies Inc.; this work was not supported by the company and was performed exclusively in his academic role. Dr Seltzer has equity in RCE Technologies. All other authors have reported that they have no relationships relevant to the contents of this paper to disclose.
